# Supplementary material for: Human-specific GAPDH qRT-PCR is an accurate and sensitive method of xenograft metastasis quantification
Source: Mol Ther Methods Clin Dev. 2020 Dec 25;20:398–408. doi: 10.1016/j.omtm.2020.12.010 (PMC7848707; doi:10.1016/j.omtm.2020.12.010)
Supplement: Document S1. Figures S1–S4 and Tables S1–S5 [file mmc1.pdf]

OMTM, Volume *20*

Supplemental Information

Human-specific GAPDH qRT-PCR is an accurate and sensitive method of xenograft metastasis quantification

Margaret L. Dahn, Cheryl A. Dean, Diana B. Jo, Krysta M. Coyle, and Paola Marcato

Supplemental Figures:

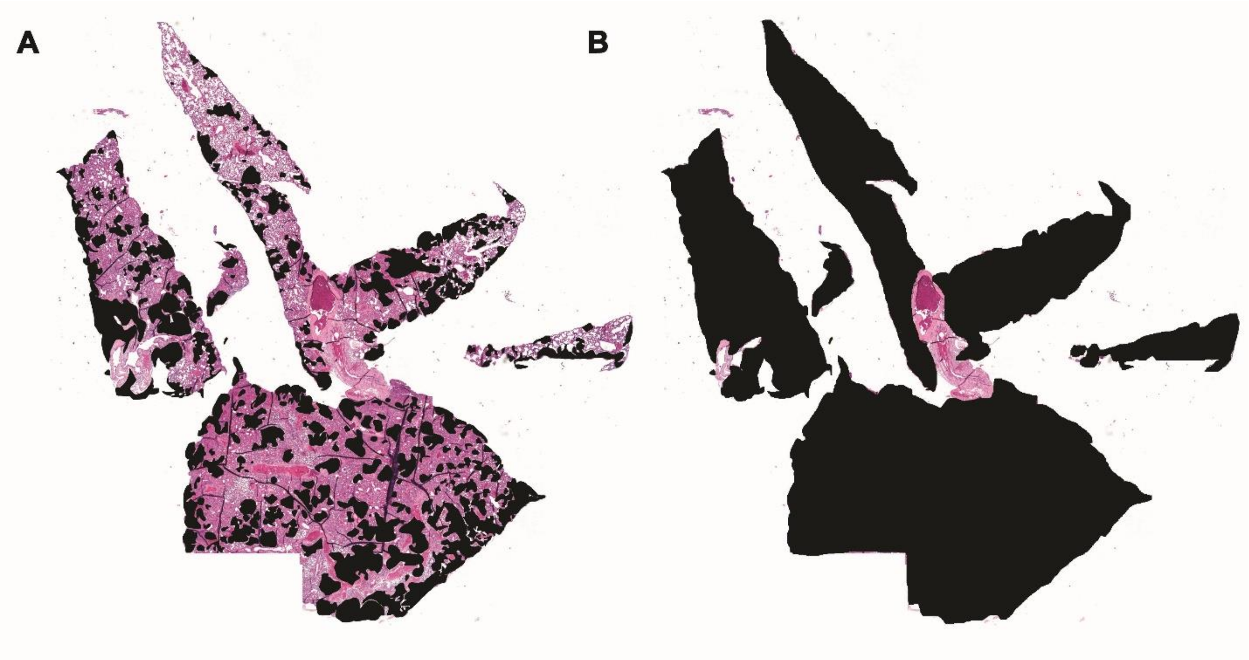

Supplemental Figure 1. Quantification of lung metastasis of H&E stained thin section using Image J. In the Image J software, the microscopically captured images were quantified of metastatic surface area by using the Freehand tool to circle and pixelate the metastatic foci **(A)**. The entire area of the lung was then selected and pixelated using the Freehand tool **(B)**, and % metastatic surface area for the thin section was calculated.

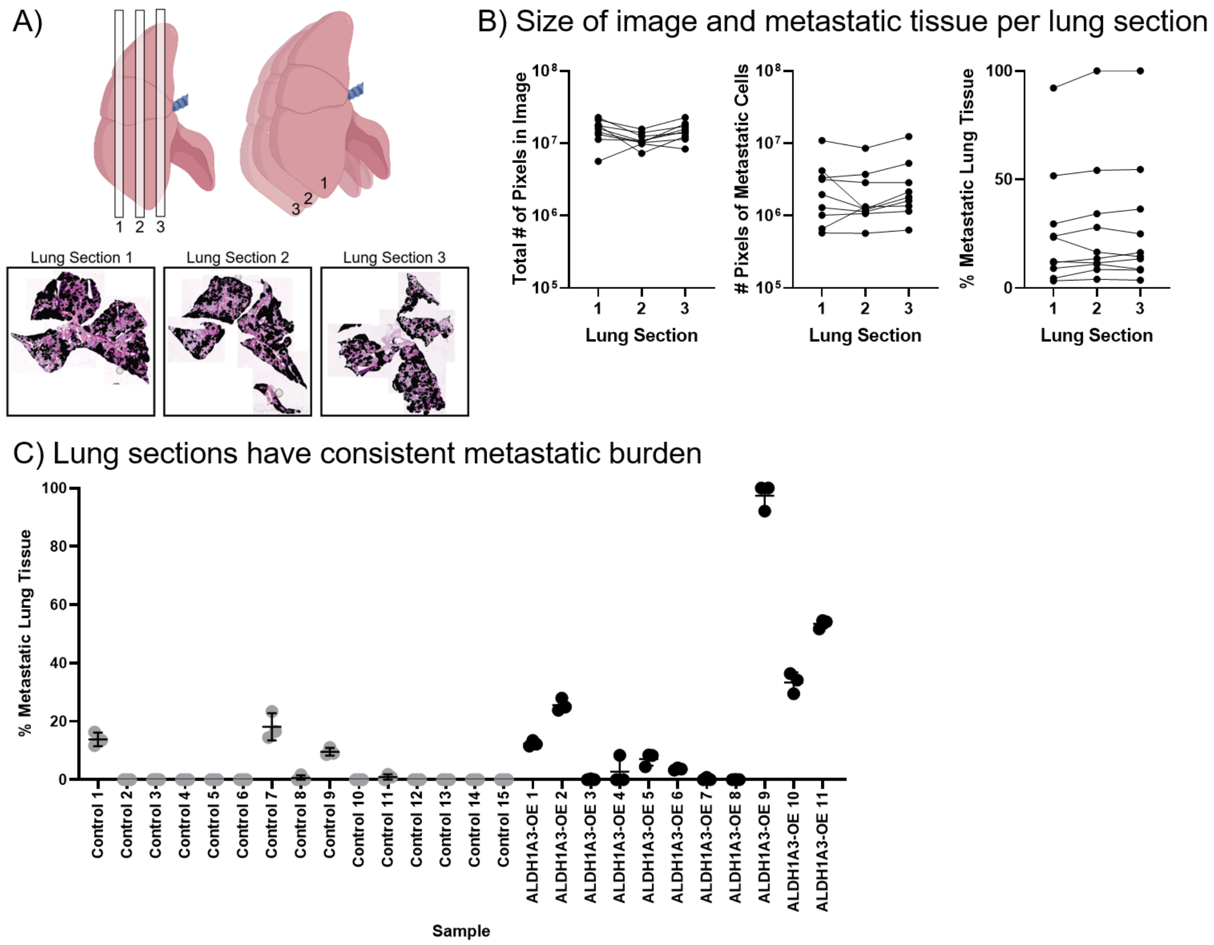

Supplemental Figure 2. Assessment of intrasample variability for metastasis quantification.

A) Three longitudinal sections were taken of each lung along the sagittal (left) or coronal (right) plane from the first quarter, middle and last quarter of the right four-lobed lung. Representative images are shown for the three resulting sections. **B)** There is low intrasample variability when comparing metastatic burden between sections. The amount of tissue assessed in each section (as determined by number of pixels within the tissue) was similar between sections and between samples (left). The amount of metastatic tissue in each section (as determined by the number of pixels within metastatic regions) was consistent between sections taken from the same sample (middle). The portion of the lung which was composed of metastatic cells is consistent across

sections taken from the same sample (right). **C)** The mean % metastatic lung tissue across the three sections for each sample; standard deviation bars.

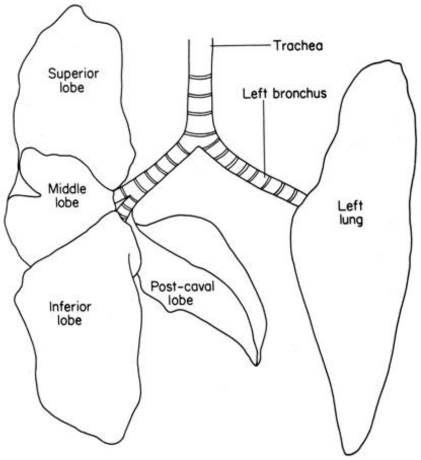

Supplemental Figure 3. Diagram of mouse lung. For consistency, the left lung lobe is always taken for the species-specific GAPDH RT-qPCR metastasis quantification method (the remainder is used for histological quantification).When mincing lung tissue in preparation for RNA extraction, utilizing two surgical blades simultaneously in a crisscross motion allows for easier/efficient mincing and dissociation of tissue.

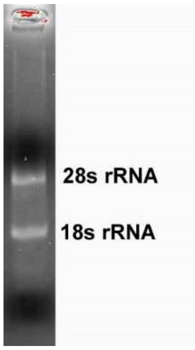

Supplemental Figure 4. RNA gel to confirm the integrity of purified RNA samples. The above image is a representative sample of purified mouse lung RNA utilizing the described method. The

visible crisp bands are 28s rRNA and 18s rRNA, which is indicative of intact RNA. Two ug of RNA (1 volume, 5μL) was added to three volumes (15μL) of RNA Sample Loading Buffer for NA electrophoresis, with ethidium bromide (50 μg/mL, Sigma-Aldrich) and heated at 65°C for 10 minutes and then kept on ice before being run on 1% agarose Tris Acetate-EDTA (TAE) gel. Molecular grade, RNase/DNase-free reagents were used and the gel apparatus was treated with RNaseZAP (Sigma-Aldrich) prior to casting.

Supplemental Tables:

Supplemental Table 1. Human and mouse GAPDH primer sequences and efficiencies.

Primer	***Forward***	***Reverse***	***Efficiency***
Mouse GAPDH (non-specific)	GCGAGACCCCACTAACATCA	GGCGGAGATGATGACCCTTT	107.4% with human cDNA, 109.8% with mouse cDNA
Human-specific GAPDH	TCAAGGCTGAGAACGGGAAG	CGCCCCACTTGATTTTGGAG	99.5%

Supplemental Table 2. ***In silico*** **analysis of human and non-specific mouse GAPDH primers.** Very wide parameters were set on NCBI Primer-BLAST for the theoretical RT-qPCR products that could be generated with a wide primer annealing temperature window (57 - 63°C) and large amplicon size range (70 - 1 000 bp) to capture any potential amplification, no matter how unlikely. Primers were run again both human and mouse reference transcriptome (NCBI RefSeq Database).

Primer	***Comparison Reference Transcriptome***	***Complementary Targets Identified***
Non-Specific mouse	Human	• No complementary sequences identified
	Mouse	• 100% fidelity for GAPDH TV1 and 2 and predicted TV1<br>• 4 mismatches on each primer to Kntc1 variants<br>• 1 mismatch on F primer to LOC115487111
Human-specific	Human	• 100% fidelity for GAPDH TV1-4<br>• 2 mismatches on F for GAPDH TV7
	Mouse	• 5 mismatches F, 4 mismatches R for MPRIP

Supplemental Table 3. Procedure for Infiltration (paraffin embedding of fixed lung tissue).

Note: All dehydration, hydration, and xylene steps are conducted in the hood for proper air ventilation. Once lung has been formalin fixed and washed in 70% ethanol, the tissue is ready to be processed for paraffin embedding. Tissues are first dehydrated, cleared with xylene and infiltrated (embedded) into paraffin wax, as shown in Supplemental Table 2. Tissues are embedding in paraffin wax using embedding rings, then placed at 4°C for 15 minutes to solidify.

DEHYDRATION	
70% ethanol	1.5 hr
95% ethanol	1.5 hr
95% ethanol	1.5 hr
100% ethanol	1.5 hr
100% ethanol	1.5 hr
100% ethanol	1.5 hr

CLEARING	
50:50 (100% ethanol:xylene)	1 hr
Xylene	1 hr
Xylene	1 hr

INFILTRATION (EMBEDDING)	
Paraffin wax (Tissue Prep, melting point 56-57C)	1 hr
Paraffin wax	1 hr

Supplemental Table 4. Procedure for H&E staining of fixed thin sections.

Note: Slides containing paraffin sections placed in a glass slide holder. Slides are first deparaffinized, then rehydrated in ethanol. Slides are then deionized in ddH_2O before H&E staining. Slides are then dehydrated in ethanol and xylene, before a cover slip is placed on top of the sections using Permount (xylene based).

DEPARAFFINIZE and REHYDRATE

Xylene	3 x 5 minutes
100% ethanol	3x 5 minutes
95% ethanol	2 x 5 minutes
70% ethanol	2 x 5 minutes

HEMATOXYLIN and EOSIN STAINING

Hematoxylin	1 x 60 dips
H2O (tap water)	1 x until excess stain is removed
Scott's H2O	1 x 30 dips
H2O (tap water)	1 x 10 dips
Eosin	1 x 20 dips
70% ethanol	1 x 30 dips
70% ethanol	1 x 30 dips

DEHYDRATION

70% ethanol	2 x 2 minutes
95% ethanol	2 x 2 minutes
100% ethanol	3 x 2 minutes
Xylene	3 x 2 minutes

Supplemental Table 5. qPCR reaction steps and temperatures.

qPCR CFX384 Steps		
1	95.0°C	0:30
2	95.0°C	0:05
3	60.0°C	0:05
	+ Plate Read	
4	Go To 2	40 more times
5	65.0 to 95.0°C	Increment 0.5°C for 0:05
	+ Plate Read	
End		
