# Supplementary material for: Human-specific GAPDH qRT-PCR is an accurate and sensitive method of xenograft metastasis quantification
Source: Mol Ther Methods Clin Dev. 2020 Dec 25;20:398–408. doi: 10.1016/j.omtm.2020.12.010 (PMC7848707; doi:10.1016/j.omtm.2020.12.010)
Supplement: Document S2. Article plus supplemental information [file mmc2.pdf]

Molecular Therapy
Methods & Clinical Development
Original Article

Human-specific GAPDH qRT-PCR is an accurate and sensitive method of xenograft metastasis quantification

Margaret L. Dahn,[1] Cheryl A. Dean,[1] Diana B. Jo,[2] Krysta M. Coyle,[1] and Paola Marcato[1,2]

[1]Department of Pathology, Dalhousie University, Halifax, NS B3H 4R2, Canada; [2]Department of Microbiology and Immunology, Dalhousie University, Halifax, NS B3H 4R2, Canada

Metastasis is the primary cause of cancer-related mortality. Experimental models that accurately reflect changes in metastatic burden are essential tools for developing treatments and to gain a better understanding of disease. Murine xenograft tumor models mimic the human scenario and provide a platform for metastasis analyses. An *ex vivo* quantitative method, gaining favor for its ease and accuracy, is quantitative reverse-transcriptase polymerase chain reaction (qRT-PCR); however, it is currently unclear how well this method correlates with gold-standard histological analysis, and its use has required detection of overexpressed exogenous genes. We have introduced a variation of the qRT-PCR method: human-specific glyceraldehyde 3-phosphate dehydrogenase (GAPDH) qRT-PCR, which allows quantification of metastasis in xenograft models without the requirement of overexpressed exogenous genes. This makes the method easily amenable to many xenograft models without alteration of the cancer cells. We determined that the method is able to detect a few human cells within abundant mouse lung tissue. Further, the human-specific GAPDH qRT-PCR is more sensitive and correlates with histological analysis in terms of determining relative metastatic burden, suggesting that human-specific GAPDH qRT-PCR could be used as a primary method for quantification of disseminated human cells in murine xenograft models.

INTRODUCTION

Metastasis is the predominant cause of cancer-related mortality.[1,2] It is a multistep process wherein cancer cells at the site of origin spread to secondary tissues and organs; the lungs are a common site of metastasis in a wide range of cancers. Understanding metastasis and the development of therapeutic agents that prevent metastasis requires *in vivo* metastatic experimental models that reflect the human scenario. Favored among researchers are murine tumor models of metastasis where cancer cells are orthotopically implanted, allowing for tumor-specific preferences for dissemination to distant sites, such as lung, brain, liver, and bone.[3] Rapid, cost-effective, and accurate measurement of lung metastasis is important to many preclinical projects.

There are numerous quantitative tools to measure metastasis in animal models, some of which are designed to mimic the clinical setting and allow for metastasis quantification in a live animal. These methods include small-animal magnetic resonance imaging (MRI), positron emission tomography (PET), and computed tomography (CT) and bioluminescence imaging (BLI) of live animals.[4–8] In the clinic, MRI is used to detect, locate, characterize, and stage cancer and assess response to treatment. In small animals, MRI offers sensitivity and specificity, due to its intrinsic spatial/temporal/contrast resolutions and adequate detectability for a tiny amount of substances, making it ideal for research.[6] Small-animal CT and PET used in combination provide both clear imaging of normal tissues and tumors and the ability to discriminate between normal and malignant tissues based on altered molecular activity.[5] BLI measures photon emission from cancer cells that are engineered to express the luciferase protein and detects the presence of these cells in organs and tissues. The luciferase reaction and production of luminescent product require live tumor cells and the luciferin substrate that relies on adequate distribution.[8] In addition to live-animal imaging, BLI can be used *ex vivo* to quantify lung metastasis in dissected lung tissue. Both MRI and BLI require specific costly equipment and facilities, which are prohibitive for some researchers. Hence, there is a need for methods that use equipment and facilities common in molecular biology laboratories.

A number of *ex vivo* methods have been developed for metastasis quantification amenable to most molecular biology laboratories. Flow cytometry analysis can be used for lung metastasis quantification. For example, cancer cells engineered to express green fluorescent protein (GFP) can be detected by flow cytometry and relative percentages of GFP^+ cells determined in harvested tissues.[9] However, this method is likely error prone as red blood cell-free single-cell suspensions need to be generated, and the cancer cells need to be tagged with or express a fluorochrome, which could alter cell behavior. Alternatively, clonogenic-based assays can be used to quantify lung metastasis.[10–13] In this technique, harvested tissues are dissociated into a single-cell suspension, plated as single cells, and cultured for at least

Received 17 October 2020; accepted 22 December 2020;
https://doi.org/10.1016/j.omtm.2020.12.010.

Correspondence: Paola Marcato, Department of Pathology, Dalhousie University, Room 11C1, 5850 College Street, Halifax, NS B3H 4R2, Canada.
E-mail: paola.marcato@dal.ca

This is an open access article under the CC BY license (http://creativecommons.org/licenses/by/4.0/).

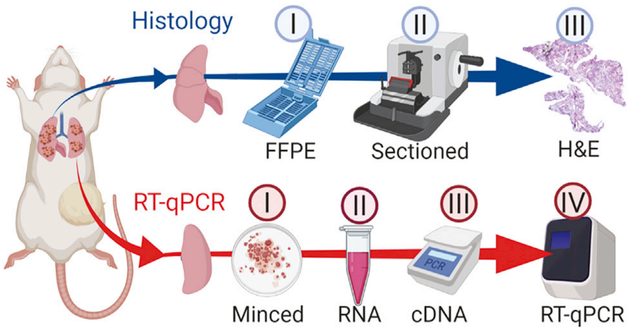

Figure 1. Schematic representation of workflow comparing quantification of lung metastasis by histological analysis of H&E-stained, fixed thin section versus human-specific GAPDH qRT-PCR

(Top) Lung processed for histology-based quantification. I, Formalin-fixed paraffin-embedded (FFPE): majority of multi-lobed lung kept in histology cassette for 24 h in formalin and then stored in 70% ethanol, followed by dehydration/clearing/embedding. II, Sectioning by microtome cutting. III, H&E staining includes deparaffinization/rehydration/staining, followed by dehydration and coverslipping. Images were captured by 2.5× magnification and metastasis quantified in ImageJ. (Bottom) Lung processed for qRT-PCR-based quantification. I, Single-lobed left lung minced/homogenized and added to II, 1 mL of TRIzol for subsequent RNA extraction. III, cDNA synthesized; IV, qPCR performed with GAPDH human-specific primers and nonspecific mouse GAPDH primers as a reference gene. Created with BioRender (https://biorender.com/).

10–14 days in a drug selection media (e.g., 4T1 cells are resistant to 6-thioguanine[14]), followed by subsequent staining and enumeration of resulting colonies. This assay requires a method of selection of cancer cells over noncancer cells, which limits the models that can be used and is potentially error prone (since it relies on the assumption that the colonies that form from the processed samples accurately represent the metastasis that was present in the tissues at the time of harvest).

Stereological analysis estimates metastasis volumes in mouse lungs by hematoxylin and eosin (H&E) staining of thin sections of cryostat-embedded lungs.[15] A variation of the stereological analysis is to perform H&E staining of thin sections of fixed, paraffin-embedded tissues and perform microscopic quantification of metastatic surface areas. This closely mimics the clinical pathological practice, and it is commonly used and is therefore often seen as the gold-standard method;[16–21] however, achieving quantitative values of metastasis by histological examination is labor intensive and requires quantification of thin sections throughout the specimen.[22]

An alternative *ex vivo* method gaining favor is based on the amplification of tumor-specific transcripts using quantitative reverse-transcriptase polymerase chain reaction (qRT-PCR). This method gives an assessment of the overall metastatic burden of the total tissue, independent of uneven distribution of metastasis in the tissue. qRT-PCR has been shown to be a sensitive method, detecting 10 cancer cells per 0.5–2 μg of lung tissue.[23] However, this was based on detection of an overexpressed transcript found in only some cell lines (i.e., the human epidermal growth factor receptor 2 [HER2], transcript in HER2-overexpressing cell line JIMT1). Similarly, others have been able to quantify lung metastasis by qRT-PCR by ectopic overexpression (OE) of a foreign gene (i.e., luciferase gene).[24] Luciferase gene expression quantification by qRT-PCR in harvested lungs was shown to be correlate with metastasis quantification by BLI and is 10 times more sensitive,[24] suggesting that qRT-PCR could be used as an endpoint readout when used in combination with BLI.

The widespread use of qRT-PCR as a technique for evaluating murine metastasis is thus limited by the requirement of a highly expressed gene that is unique to the cancer cells and the lack of comparison between metastasis quantification by qRT-PCR and a well-accepted method (e.g., histological analysis of H&E-stained thin sections). Herein, we describe our method of quantifying expression of an abundant human and mouse glyceraldehyde 3-phosphate dehydrogenase (GAPDH) transcript by qRT-PCR utilizing validated human-specific primers. We demonstrate that human-specific GAPDH qRT-PCR is highly sensitive, discriminating between transcripts of human and mouse origin with high specificity. The method reliably detects 100 human cells in a mouse lung lobe (∼70 mg tissue). Human-specific GAPDH qRT-PCR was successfully utilized to detect increased metastatic burden imparted by aldehyde dehydrogenase 1A3 (ALDH1A3) expression in MDA-MB-231 breast cancer cells and demonstrated that MDA-MB-231 cells are more metastatic than MDA-MB-436 cells. A direct comparison of lung metastasis quantified using human-specific GAPDH qRT-PCR versus histological quantification of H&E-stained thin sections and found the two methods highly correlative, with the former being more sensitive.

RESULTS

Herein, we compare two methods for quantification of lung metastasis in a murine xenograft tumor model: the gold-standard histological-based method of quantifying percent metastasis of sectioned and stained fixed lung tissue versus a qRT-PCR-based method of quantifying levels of human cancer cell-specific transcripts in RNA extracted from murine lung tissue. We have introduced a variation of the qRT-PCR method that will allow accurate and sensitive quantification of metastasis without the requirement of OE of exogeneous genes in the context of xenograft models (i.e., human-specific GAPDH qRT-PCR). In Figure 1, we outline the workflow and how we will directly compare these two methods using the same experimental lung specimens.

Histological quantification of lung metastasis

To compare the two methods of metastasis quantification, we established a tumor xenograft model with previously demonstrated differing levels of lung metastasis to better test the sensitivity of the methods. We utilized MDA-MB-231 cells, with or without ALDH1A3 OE,[21] and MDA-MB-436 cells. By using histological quantification methods, we previously determined that when these cells are orthotopically implanted in the mammary fat pads of non-obese diabetic (NOD)/severe combined immunodeficiency (SCID) mice, MDA-MB-231 lung metastasis occurs in approximately 50 days and that ALDH1A3 OE increases the metastatic burden.[21]

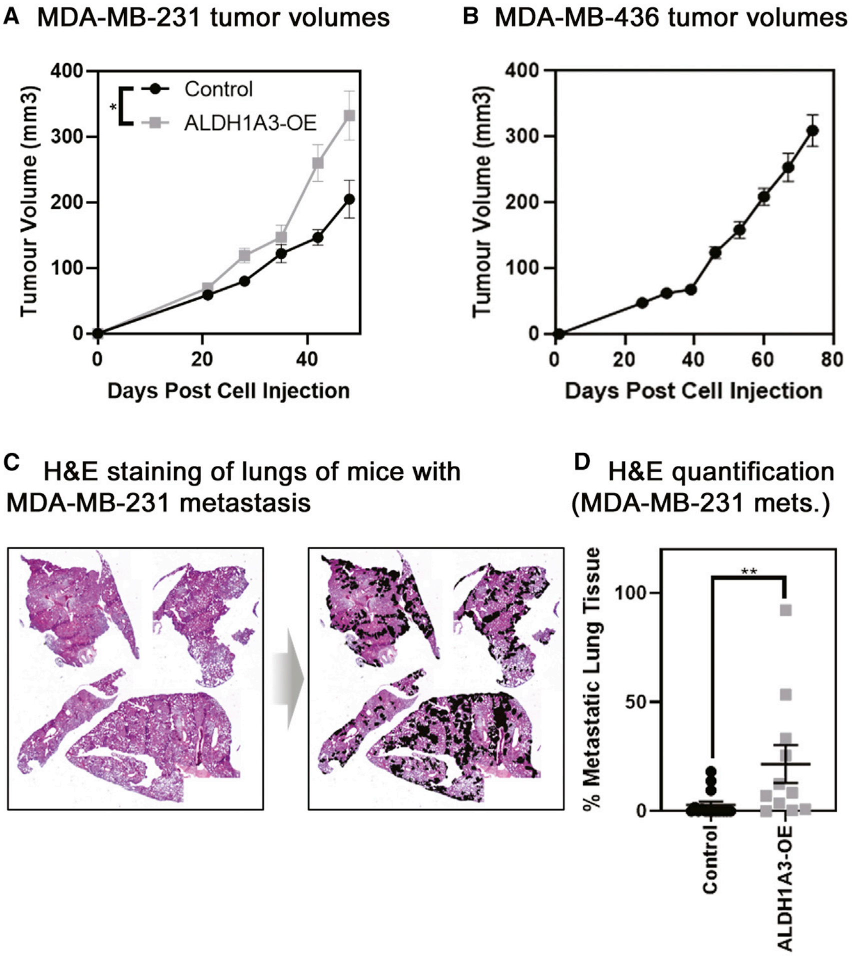

Figure 2. Tumor volumes and histological quantification by H&E staining of fixed thin sections demonstrate that ALDH1A3 expression increases metastasis to the lungs of orthotopically established MDA-MB-231 tumors

(A) ALDH1A3 overexpression increases MDA-MB-231 tumor volume and control (n = 15; ALDH1A3 overexpression n = 11, SEM error bar; one-tailed t test performed on final volume measurement). (B) MDA-MB-436 tumor volume (n = 13, SEM error bars). (C) Light microscopy image of H&E-stained lung section from mice that had MDA-MB-231 tumors; example from ALDH1A3 overexpression tumor-bearing mouse with metastatic nodes outlined. (D) Histology-quantified MDA-MB-231 lung metastasis (median, SEM error bars; Mann-Whitney test); *$p < 0.05$; **$p < 0.01$; ***$p < 0.001$.

Here, we terminated the experiment on day 49 for the MDA-MB-231 tumors, noting a significant increase in tumor volume upon ALDH1A3 OE (Figure 2A), and day 74 for the slower-growing MDA-MB-436 tumors (Figure 2B). We determined the metastatic burden resulting in each of the MDA-MB-231 experimental mouse lungs using the gold-standard method of quantification (histological analysis of H&E-stained lung thin sections spaced throughout the fixed lung paired with ImageJ analysis of captured images; Figures 2C and S1).

Notably, others have reported that quantitative assessment of the metastatic load is possible by counting the average number of micrometastases in ten histological thin sections within the middle of the lung.[25] Jojovic and Schumacher[25] did note that there are fewer micrometastases at the periphery of the lung and hence, proposed subtracting 20% from the number of micrometastases averaged from the 10 middle thin sections. We instead quantified the average percentage of metastatic surface area in three vertical, thin sections taken at the first quarter, middle, and last quarter of the lung (Figure S2). To assess the reliability of this quantification method, we plotted the percentage metastasis of each thin section and noted very little variability within a lung specimen, regardless of whether the percentage of metastasis was determined from the first quarter, middle, or last quarter of the lung (Figure S2). This suggests that quantifying three spaced sections covering different zones in the lung for average metastatic area is sufficient to give a reliable indication of the overall metastatic load. The high level of agreement between the histological analysis and qRT-PCR method also suggests that the histological analyses we preformed reflect the metastatic load of the individual specimens. It is also possible that our method of quantifying metastatic surface area versus enumerating micrometastases in a thin section[25] may give less interslide variability.

Our histological metastasis quantification established a reference for later comparison with the qRT-PCR method and also confirmed that the MDA-MB-231 cells had metastasized to the lungs in these specimens and that ALDH1A3 OE increased metastasis (Figure 2D). With the use of the histological analysis method, we detected lung metastasis in 5/15 of the control and 11/11 ALDH1A3-OE lung specimens (16/26 lungs total). In the 16 positive metastatic tissue specimens, metastasis ranged from 0.07% to 92.14%. Therefore, histological quantification of the lungs discriminates differences in metastatic burden between different experimental conditions, and the limit of detection of metastasis was 0.07% of total lung tissue (averaged over 4 lung lobes and three thin sections/specimen).

Identification of human-specific GAPDH primers that discriminate between mouse tissue and human cells

High sensitivity in qRT-PCR-based methods of lung metastasis quantification is more likely achievable if detecting abundant cancer cell-unique transcripts. GAPDH is among the most abundantly expressed transcripts; hence, if human-specific primers to GAPDH that discriminate between human and mouse transcripts in qRT-PCR

are successfully designed, then it could be a highly sensitive method for detection of disseminated human cancer cells in murine xenograft models. Further, it would preclude the requirement of introduction of a foreign gene and be amenable to all xenograft models, eliminating the need for a cell line-specific overexpressed gene (e.g., HER2).

With the use of National Center for Biotechnology Information (NCBI) Primer-Basic Local Alignment Search Tool (Primer-BLAST), we designed primers to unique regions of the human GAPDH transcript that would generate amplicons of 86 bp for the human GAPDH transcript (Table S1). The primers have a theoretical annealing temperature of 60°C. We performed an *in silico* analysis of the specificity of the human GAPDH primers against the human and mouse transcriptome (Table S2). The *in silico* analysis of primer specificity suggests that the primers should be species specific in terms of detecting GAPDH mRNA, but this needs to be tested.

To confirm the specificity of the primers, we used the human GAPDH primers in qRT-PCR experiments against RNA isolated from cultured MDA-MB-231 cells and naive NOD/SCID mouse lung (harvested from a mouse that had no exposure to human cells). We similarly used the primers designed against mouse GAPDH with the two different templates. Serial dilutions of the cDNA templates in the qPCR reactions revealed that our human GAPDH primers were highly specific to the template of human origin (MDA-MB-231 cells) (Figure 3A). There was no product when the human GAPDH primers were used in qPCR reactions with a mouse lung cDNA template. In contrast, the primers designed to quantify mouse GAPDH amplified a product to cDNA templates of both human (MDA-MB-231 cells) and mouse origin (mouse lung tissue; Figure 3A). The data with the mouse primers reinforce the notion that all primers made to a certain species need to be validated in qRT-PCR to confirm the primer specificity for the template of said species. The melting curves confirmed that only one product was consistently made in the qPCR reactions, even in reactions with a highly diluted template (Figure 3B). With the use of the amplification data from the serially diluted cDNA qPCRs (Figure 3A), we generated standard curves (Figure 3C). This revealed that the primers were highly efficient over a wide range of cDNA template concentrations (Figure 3C). Finally, we tested the primers in side-by-side reactions with three different samples of concentrated MDA-MB-231 and mouse lung (Figure 3D). This confirmed that the human-specific GAPDH primers generate amplicons from templates of human origin and not mouse, whereas the mouse primers are nonspecific in terms of species (Figure 3D). Together, these data validated that the human GAPDH primers are highly human specific and efficient in terms of generating a product by qRT-PCR (Figure 3). Importantly, our metastasis quantification assay requires that only the human GAPDH primers be highly specific to human cDNA template; therefore, we proceeded to the next validation step.

Human-specific GAPDH primers identify 100 human cells in a mouse lung lobe

Having identified human GAPDH primers with high specificity for transcripts of human origin, we next determined the detection and specificity limit of the human GAPDH primers for human GAPDH transcripts in the context of abundant mouse transcripts. For this analysis, we added known amounts of serially diluted MDA-MB-231 and MDA-MB-436 cells (0; 10; 100; 1,000; 10,000; 100,000; and 1,000,000 cells) into tubes containing a naive NOD/SCID lung lobe. We then purified the total RNA and performed qRT-PCR on the samples using the human-specific GAPDH primers and nonspecific mouse GAPDH primers as a reference gene. The resulting CT values demonstrate the high specificity of the human-specific GAPDH primers; in the context of abundant mouse RNA, the human GAPDH transcript was detectable when as few as 100 MDA-MB-231 or MDA-MB-436 cells were spiked into the mouse lung lobe sample (Figure 4A). The standard curve revealed that quantification of human GAPDH transcript in the context of abundant mouse lung RNA was linear between the range of 100 and 1,000,000 MDA-MB-231 or MDA-MB-436 cells (Figure 4B). Together, these data suggest that the method is similarly sensitive at detecting human cancer cell lines of different origins (e.g., MDA-MB-436 or MDA-MB-231) in the context of abundant mouse tissue.

Human-specific GAPDH qRT-PCR is highly sensitive and an accurate method of xenograft lung metastasis in comparison to histological quantification

Having established the dynamic range and specificity of the human-specific GAPDH qRT-PCR, we next quantified the lung metastasis in the left lobe of the harvested lung from the MDA-MB-231 xenograft experiment (with or without ALDH1A3 OE; Figure 2A) or MDA-MB-436 cells. With the use of the standard curve (Figure 4B), this revealed the number of MDA-MB-231 cells present in each lung lobe sample. All of the experimental lung samples fell within the standard curve (Figure 5A) and were therefore usable samples for metastasis quantification. As expected, based on the histological analysis of the same lung specimens (Figure 2), human-specific GAPDH qRT-PCR also showed that ALDH1A3 OE resulted in increased metastasis to the lungs of the mice (Figure 5B).

With the use of the human-specific GAPDH qRT-PCR method, we also assessed lung metastasis in the MDA-MB-436 orthotopic tumor-implantation model. Notably, MDA-MB-436 cells have been previously characterized as nonmetastatic or having low metastatic activity in comparison to MDA-MB-231 cells.[26,27] We therefore used the MDA-MB-436 tumor xenograft model to determine the limits of the human-specific GAPDH-qPCR methods for detecting disseminated cancer cells and if the method would demonstrate that MDA-MB-436 cells are less metastatic than MDA-MB-231 cells. We harvested the lungs from NOD/SCID mice that had been implanted with MDA-MB-436 tumors in their mammary fat pads for 74 days (Figure 2C) and performed human-specific GAPDH qRT-PCR on the lungs. This revealed a range of 0 to 102 MDA-MB-436 cells in the lungs of the mice (Figure 5C), which is orders of magnitude less than the number of MDA-MB-231 cells that was detected in the MDA-MB-231 xenograft experiment (Figure 5B). These results further substantiate the human-specific GAPDH qRT-PCR as a general method of quantifying metastasis in xenograft tumor models, capable of detecting a few disseminated human cells in a mouse organ/tissue.

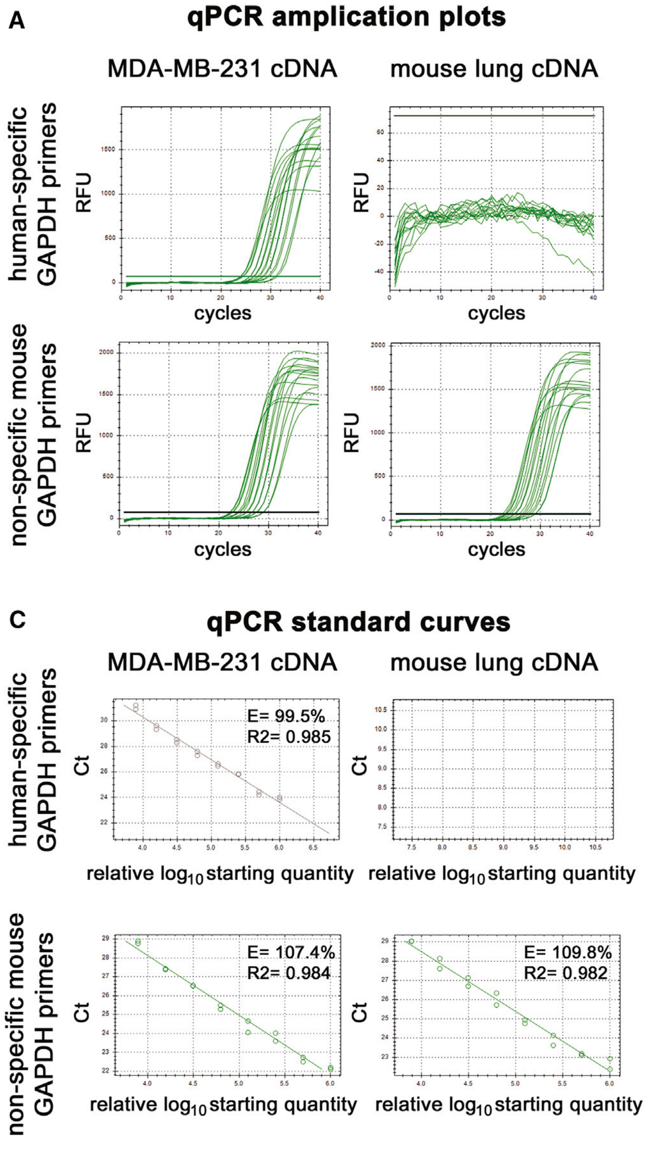

Figure 3. Validation that the human GAPDH primers are efficient human-specific primers

(A and B) The qPCR amplification curves (A) and melt curves (B) generated by the human and nonspecific mouse GAPDH primers using a 2-fold serially diluted cDNA template from RNA extracted from MDA-MB-231 cells and naive NOD/SCID mouse lung. MT, melting temperature in Celsius at the peak. (C) Standard curves were generated using the qPCR amplification from (A), and the starting quantities of the 2-fold diluted cDNA are arbitrary, with the most concentrated samples set at the default setting of 1,000,000. E, efficiency; R2, the square of the correlation (the coefficient of determination). (D) Three different concentrated MDA-MB-231 and naive NOD/SCID mouse lung RNA samples were analyzed via qRT-PCR with the human and nonspecific mouse GAPDH primers.

Next, we assessed that human-specific GAPDH qRT-PCR versus the gold-standard histological method of quantifying lung metastasis is highly correlative (Figure 6). With the use of this qRT-PCR method, we found between 71 and 219,750 MDA-MB-231 cells in every lung lobe sample; in comparison to the histological method, no metastasis was detectable in 10 lungs (Figure 6A). This demonstrates that human-specific GAPDH qRT-PCR is more sensitive than histological quantification of lung metastasis in the xenograft tumor model. The correlation analysis between the GAPDH qRT-PCR and gold-standard histological methods demonstrates that human-specific GAPDH qRT-PCR is an accurate method of lung metastasis (Figure 6B). Of note is that the correlation is negatively impacted by samples with less than 1% lung metastasis due to those samples falling near the detection limit of metastasis for the histological quantification method.

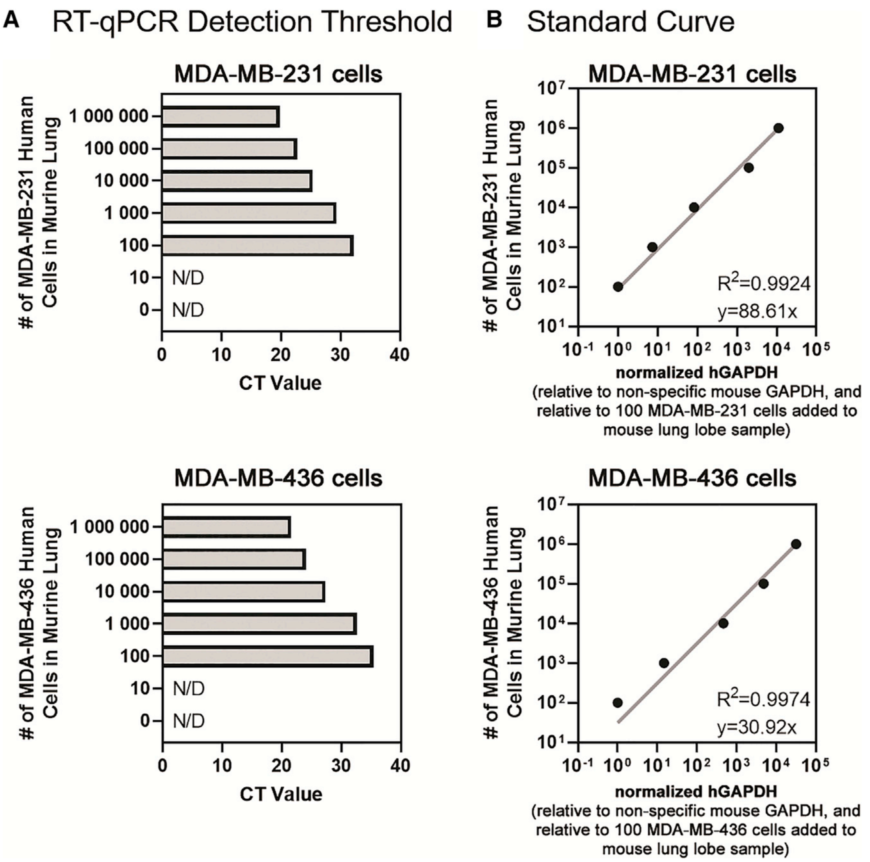

Figure 4. The detection limit of human-specific GAPDH qRT-PCR is 100 human cells per mouse lung lobe, and the standard curve is a linear range between 100 and 1,000,000 human cells per mouse lung lobe

(A) Increasing numbers of known MDA-MB-231 cells (top) or MDA-MB-436 cells (bottom) were added to naive NOD/SCID lung lobe samples and total RNA extracted and the detection limit of human-specific GAPDH primers determined by qRT-PCR. N/D, not detected. (B) Standard curve of the number of MDA-MB-231 cells (top) or MDA-MB-436 cells (bottom) added in the naive NOD/SCID lung lobe plotted against the relative amount of human GAPDH transcript detected by qRT-PCR. The normalized human GAPDH (detected with human-specific primers) per sample was made relative to the total GAPDH (detected with nonspecific mouse primers, ΔΔCt) and made relative to the 100 MDA-MB-231 cells added to a naive mouse lung lobe sample.

Human-specific GAPDH qRT-PCR is a relatively cost effective and a rapid method of xenograft lung metastasis

Based on our comparison of the various methods of murine metastasis quantification, human-specific GAPDH qRT-PCR presents several advantages, including its relative low cost and quick method. A time and cost comparison of histological/H&E thin-section staining versus human-specific GAPDH qRT-PCR methods shows that the former is much more labor intensive. To quantify lung metastasis by the histological approach, it required approximately 120 h of technician time to perform lung dissection, tissue embedding and sectioning, H&E staining, image capturing, image stitching, and ImageJ analysis. In contrast, human-specific GAPDH qRT-PCR on the same number of samples required approximately 25 h of technician time to perform lung dissection, RNA extraction and quantification, cDNA generation, and qRT-PCR analysis. This equates to approximately 21% of the time to obtain quantification of lung metastasis by qRT-PCR versus histological analysis, which translates to having the results in less than 1 week using the qRT-PCR method and nearly 1 month using the histological quantification method. Therefore, despite the perhaps increased cost of reagents for qRT-PCR over histological analysis, when the personnel time is considered, qRT-PCR is the more economical and efficient option.

DISCUSSION

Herein, we have described and directly compared the use of human-specific GAPDH qRT-PCR and histological analysis of H&E-stained, fixed thin sections to quantify lung metastasis in murine xenograft tumor models. We provide instruction for dissecting and sectioning mouse lung tissue, utilizing the left lobe for qRT-PCR, and leaving the 4 right lobes for alternate analyses if required; our procedure utilized the 4 remaining lobes for histological quantification. Several modifications have been provided for lung dissection, RNA extraction, human-specific primer generation, and qRT-PCR that differentiate and improve upon methods reported previously.[5,23]

We acknowledge that other methods, including continuous monitoring of live animals (possible with BLI, PET, CT, and MRI) and end-point analyses (e.g., clonogenic assays, flow cytometry, and stereological analyses), are also effective for detecting/quantifying metastasis, each having its own strengths and limitations. Stereological and histological analyses by H&E staining closely mimic the clinical pathological practice and are therefore commonly used to quantify lung metastasis, with the latter being the gold standard.[28,29] However, histological examination of the whole organ (i.e., multiple sections from throughout the fixed tissue) is necessary to avoid variability due to sampling error and is largely qualitative unless an imaging software for quantification is also incorporated.[22] Therefore, it becomes a labor-intensive procedure. In addition, the smallest metastases that can be observed reliably contain ∼10 cells in a foci.[15]

qRT-PCR has proven to be a sensitive measure to quantitatively detect cancer cell transcripts (exogenously introduced or overexpressed in a specific cancer cell line) within the mouse lung tissue.[23,24]

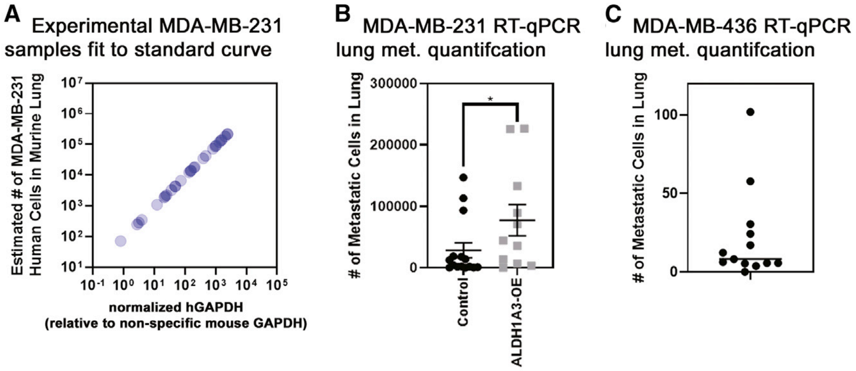

Figure 5. Human-specific GAPDH qRT-PCR quantification of lung metastasis demonstrates that ALDH1A3 expression increases metastasis to the lungs of orthotopically established MDA-MB-231 tumors

(A) The number of MDA-MB-231 cells per lung lobe in the experimental samples is determined by using the standard curve generated in Figure 4B. Furthermore, the normalized human GAPDH (detected with human-specific primers) per sample was made relative to the total GAPDH (detected with nonspecific mouse primers, ΔΔCt). (B) The number of MDA-MB-231 cells/lung lobe in control (n = 15) versus ALDH1A3 overexpression (n = 11) samples is compared by human-specific GAPDH qRT-PCR median, SEM error bars; Mann-Whitney test; $^*p < 0.05$; determined using the standard curve of known MDA-MB-231 cells/lung lobe. (C) The number of MDA-MB-436 cells/lung lobe in samples is determined by human-specific GAPDH qRT-PCR using the standard curve of known MDA-MB-436 cells/lung lobe. The human GAPDH per each sample was made relative to the total GAPDH detected by nonspecific mouse GAPDH primers.

Although qRT-PCR lacks the ability to differentiate between living and dead tumor cells, it provides an accurate and sensitive method for quantification of lung in murine metastasis tumor models. Human-specific primers have been utilized in qPCR to quantify mouse cell contamination in human xenografts.[30] Alcoser et al.[30] generated prostaglandin E receptor-2 (PTGER2) human-specific primers to detect contaminating mouse DNA in multiple tumor types. Here, we performed human-specific GAPDH qRT-PCR on purified RNA instead of qPCR on purified DNA, as performed by Alcoser et al.[30]. The benefit of human-specific GAPDH qRT-PCR of purified RNA over human-specific qPCR of a gene (e.g., PTGER2)-purified DNA is the much greater template copy number in the former method.

It is noteworthy that qPCR of Alu elements, with over 50,000 copies in the human genome, overcomes the copy number limitation associated with gene-specific PCR.[31] Campbell et al.[31] were able to identify 1 MDA-MB-231 cell among 1,000,000 mouse cells and quantify metastasis of MDA-MB-231 cells to the mouse bone by performing Alu qPCR on DNA purified from the tissue samples. Therefore, human-specific GAPDH qRT-PCR of purified RNA is comparable to Alu qPCR of purified DNA for detecting a few human cancer cells in abundant mouse tissue. Depending on which type of sample is available, RNA or DNA, a researcher may choose to perform human-specific GAPDH qRT-PCR or Alu qPCR,[31] respectively. Our method is perhaps the preferred method for researchers with only RNA samples or for those who may want to quantify metastatic burden and expression of other murine genes using the same RNA/cDNA sample.

To our knowledge, no other groups have utilized human-specific primers designed to unique regions of abundant human transcripts to quantify lung metastasis in murine xenograft tumor models utilizing RNA samples. We demonstrate that human-specific GAPDH qRT-PCR is highly sensitive and an accurate method of detecting xenografted cancer cells that have disseminated in mice. Importantly, this method precludes the requirement of the introduction of a foreign gene or a gene that may be overexpressed in only some cancer cell lines (e.g., HER2[23]). This makes human-specific GAPDH qRT-PCR amenable to all xenograft models without consideration for identifying an overexpressed gene or manipulation of the cell line. Perhaps the best indicator of increased sensitivity of the human-specific GAPDH qRT-PCR is the detection of cancer cells in 10/25 experimental samples that were assessed as lacking metastatic cells by H&E staining of fixed thin sections.

In addition to increased sensitivity and specificity, other advantages of this qRT-PCR method include its usability in conjunction with other quantification methods, such as an end-point assay, when paired with methods such as MRI/BLI quantification of metastasis of live animals. qRT-PCR is also more time/cost effective than many other methods. Furthermore, the method lends itself to quantification of disseminated cancer cells in other tissues besides the lung. For example, it could be used to detect the presence of cancer cells in the lymph node, liver, or brain and quantify circulating tumor cells. When applying this method to other organs and tissues, the size of the organ and tissue needs to be considered. We found that we could isolate intact RNA from ∼70 mg of the minced lung lobe in 1 mL of TRIzol; however, if the mass of the organ/tissue of interest is greater than 70 mg, then we recommend mincing the entire tissue. First, mix the minced tissue to avoid sampling error, and then taking a consistent amount (between 5 and 70 mg) of the minced tissue for RNA extraction.

We propose that qRT-PCR analysis can be performed in lieu of the other well-accepted metastasis quantification models. Importantly, the demonstration here that human-specific GAPDH qRT-PCR correlates highly with the gold-standard histological method is critical if the qRT-PCR method is to be used with confidence over other accepted metastasis quantification methods. Furthermore, our inclusion here of the ALDH1A3 OE model and MDA-MB-231 versus MDA-MB-436 models was to test the capacity of the qRT-PCR method for discriminating between different conditions with varying

A H&E and RT-qPCR MDA-MB-231 metastasis quantification numbers

Tumour Type	Sample	RT-qPCR (# of Metastatic Cells)	H&E (% Lung Tissue)
Control	1	91 694	13.86
	2	1 093	0.00
	3	2 235	0.00
	4	245	0.00
	5	2 029	0.00
	6	282	0.00
	7	139 997	18.16
	8	17 569	1.73
	9	117 518	9.58
	10	1 878	0.00
	11	12 221	0.91
	12	14 038	0.00
	13	4 268	0.00
	14	71	0.00
	15	18 562	0.00
ALDH1A3-OE	1	72 191	12.41
	2	131 997	25.58
	3	6 568	0.34
	4	42 408	8.41
	5	34 968	7.10
	6	13 511	3.67
	7	3 247	0.83
	8	349	0.07
	9	213 985	92.14
	10	86 937	33.38
	11	219 750	53.50

B Correlation between H&E and RT-qPCR quantification of MDA-MB-231 metastasis

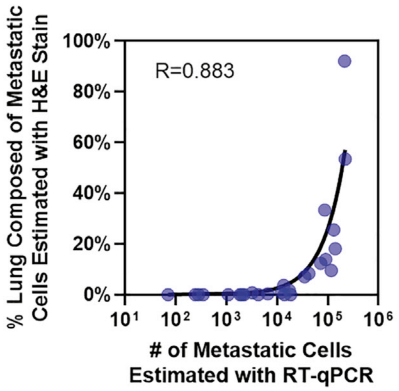

Figure 6. Human-specific GAPDH qRT-PCR correlates with and is more sensitive than histological quantification of lung metastasis by H&E staining of fixed thin sections

(A) Summary of the lung metastasis quantified in all of the experimental samples determined by human-specific GAPDH qRT-PCR (left, number of MDA-MB-231 cells/lung lobe) versus histological quantification by H&E staining of thin sections (right, percentage of MDA-MB-231 metastasis area averaged over 4 lobes of lung tissue). (B) The MDA-MB-231 lung metastasis values determined in every experimental sample by human-specific GAPDH qRT-PCR (x axis) versus histological quantification of H&E-stained, fixed thin sections (y axis) are plotted, and the Pearson correlation (R) is calculated.

metastatic burden. Both the human-specific GAPDH qRT-PCR method and the histological method similarly indicated that ALDH1A3 OE in MDA-MB-231 cells increased lung metastasis ($p < 0.05$). Now that we have validated this method, we could use this method to answer other metastasis questions. For example, we could utilize human-specific GAPDH qRT-PCR to determine if ALDH1A3 OE will enable MDA-MB-436 cells to significantly metastasize to the lungs. This is currently unknown. Hence, these comparative method analyses will be valuable for other researchers considering qRT-PCR as a primary method of metastasis quantification. qRT-PCR as a method of metastasis burden in murine xenograft models may be best suited for research groups with limited access to certain types of equipment, with time and budget constraints.

In conclusion, the data presented suggest that human-specific GAPDH qRT-PCR provides an efficient, sensitive, and cost-effective method of detecting human tumor-derived mRNA within murine tissues. The protocol should be considered as a primary method of metastasis quantification when utilizing murine xenograft models.

MATERIALS AND METHODS

Cell lines

MDA-MB-231 and MDA-MB-436 cells were obtained from the American Type Culture Collection and cultured at 37°C, 5% CO_2 incubator. MDA-MB-231 cells were cultured in Dulbecco's modified Eagle's medium (DMEM; Thermo Fisher Scientific, Life Technologies; Cat#12430062), supplemented with 10% fetal bovine serum (FBS; Thermo Fisher Scientific, Life Technologies; Cat #12483020) and 1× antibiotic-antimycotic (AA; Thermo Fisher Scientific, Life Technologies; Cat #15240062), and cultured at. MDA-MB-436 cells were grown in Leibovitz's medium (L-15; Invitrogen), supplemented with 10% FBS, 1× AA, 10 μg/mL human insulin (Sigma), and 16 μg/mL L-glutathione (Invitrogen). ALDH1A3 OE and control MDA-MB-231 clones were previously generated[21] and maintained in media supplemented with 0.25 μg/mL puromycin (Sigma-Aldrich; Cat #P8833).

Murine xenograft tumor models

A xenograft model was utilized as previously described.[21] Briefly, groups of 8- to 9-week-olid NOD/SCID female mice (Charles River) were orthotopically injected in the mammary fat pad #4 with 2×10^6 cells of either ALDH1A3 OE or vector control MDA-MB-231 cells or with 5×10^6 MDA-MD-436 cells (admixed with high-concentration Matrigel; Becton Dickinson [BD]; Cat #354262). The cells were injected within the two groups, alternating up to 5 animals, to minimize any variability due to time of injection. All of the animals were injected within the same hour. All tumor measurements were completed at the same time (within 1 h). Tumor measurements were always completed by the same person (blinded to the animal's identification) to minimize variability due to slight differences in measuring techniques. The animals had ear holes for individual identification. No animals were excluded from the experiment, as all animals bore tumors at the first measurement time point. Mice bearing MDA-MB-231 of MDA-MB-436 tumors were euthanized in in a CO_2 chamber under full anesthesia (unconscious) by isoflurane inhalation on day 49 (MDA-MB-231 experiment) or 74 (MDA-MB-436 experiment) post-cancer cell implantation. The lungs were then harvested. All animal experiments comply with the standards set by the Canadian Council on Animal Care (CCAC) and were performed according to a protocol approved by Dalhousie University's Committee on Laboratory Animals (protocol 19-013). Notably, as per the CCAC guidelines, no tumor exceeded the

17-mm diameter in any direction and were all less than 10% of the animal's body weight. The harvested samples for metastasis quantification were assigned numbers and blinded.

Mouse dissection to harvest lungs

Methods for mouse dissection have been previously published in detail;[23] however, we have made some modifications to the protocol, incorporating details described by Sleigh et al.[32] for removing the pelts. Mice were doused with 70% ethanol to allow for ease of cutting and to sterilize mouse. With sterile dissecting scissors, a small incision was made in the dorsal skin at the level of the hips and continued horizontally around the whole mouse. The pelt was pulled up and over the head of the mouse to expose the chest and rib cage. The peritoneum was cut just under and across the whole rib cage and then vertically up with middle of the rib cage. Scissors were then used to cut open the rib cage to expose lungs. The lungs were carefully removed using scissors and forceps. Thymus, heart, efferent and afferent blood vessels, trachea, and connective tissue were removed from the lung tissue. The entire left lung lobe was separated from the rest of the lung tissue (Figure S3). On average, the left lobe weighs 70 mg. The lungs were rinsed with phosphate-buffered saline (PBS; pH 7.4) to remove surface blood, which can add difficulty to histological processing.

Histological quantification of lung metastasis by thin sectioning and H&E staining

The majority of the lung tissue not utilized for qRT-PCR (4 smaller right lobes; superior, middle, inferior, and postcaval; Figure S3) was placed into a cassette and immersed in a 10% acetate-buffered formalin solution (0.2 L 37% formaldehyde, 1.8 L distilled H_2O, and 46.1 g Na acetate-$3H_2O$) for 24 h for fixation. The tissue was then rinsed 3 times in 70% ethanol and stored in 70% ethanol until paraffin embedding. For infiltration (embedding), tissues were first dehydrated, cleared with xylene, and infiltrated (embedded; Table S3). All dehydration, hydration, and xylene steps were conducted in a fumehood for proper air ventilation. Tissues were embedded in paraffin wax using embedding rings, then placed at 4°C for 15 min to solidify. Thin sections (5 μm) were cut using a fully automated microtome (Leica; RM2255). Cut sections were placed in a 42°C water bath and put on Fisherbrand Superfrost Plus Microscope Slides (Fisher Scientific; Cat #22-037-246). Slides were dried in a 37°C oven overnight before staining with H&E.

Slides containing paraffin sections were placed in a glass slide holder. Slides were first deparaffinized and then rehydrated in ethanol (Table S4). Slides were then deionized in H_2O before staining (Table S4) and then dehydrated in ethanol and xylene in advance of coverslip placement. Before a coverslip was put on the slide, a small drop of Permount (xylene based) was put on the coverslip, the coverslip was angled so that it could gently fall onto the slide, and then forceps were used to help guide coverslip in place, allowing the Permount to cover the entire section and to force out air bubbles. Once slides were mounted, they were left to dry overnight in the fumehood.

Images of three sections per block (1 from the first the quarter of block, 1 from the middle of block, and 1 from the last quarter of block) were captured on the Zeiss Axio Imager Z1 with color and monochrome camera at 2.5× magnification. To capture the entire lung section, each slide had 4−12 images captured (depending on size of tissue). Images were stitched together using Adobe Photoshop and then imported into the ImageJ program for metastasis quantification. Each metastatic colony on the lung was freehand circled and pixelated, then the entire area of the lung was freehand circled and pixelated, and percent metastasis was calculated (Figure S1). The total percent metastasis per lung was calculated by averaging percent metastasis of the three quantified thin sections.

Lung processing for RNA isolation and RNA extraction

The harvested left lung lobe (Figure S3) was placed into a Petri dish and minced with surgical blades. Please note that we did not weigh every left lung lobe (which, on average, weighs 70 mg) but did take the entire left lung lobe for RNA processing for every specimen. Other previously described protocols for RNA extraction require additional dissociation and digestions steps;[23,24] however, our protocol includes some modifications, where pure, high-quality RNA can be extracted from lung tissue pieces that are preserved in TRIzol (Thermo Fisher Scientific, Life Technologies; Cat #15596018). Minced pieces were transferred to a microfuge tube, and 1 mL of TRIzol was added. The tube was vortexed to ensure the minced pieces were dispersed and immersed in TRIzol. At this step, tubes can be flash frozen and stored in a −80°C freeze for later RNA isolation.

Additionally, a naive mouse lung lobe was spiked with increasing numbers of MDA-MB-231 cells to generate a standard curve for the qRT-PCR-based method. Six tubes containing minced lung tissue and 1 mL of TRIzol were prepared, and then 0; 10; 100; 1,000; 100,000; or 1,000,000 MDA-MB-231 or MDA-MB-436 cells were added to the tubes in 100 μL of PBS.

To prevent RNA degradation, it is critical to wear gloves when purifying RNA and handling RNA samples. It is also essential to use RNase/DNase-free certified solutions, and plastics and pipette tips need to have filters. Tubes containing TRIzol and tissue were thawed and vortexed until minced tissue was dispersed. In a modification from the manufacturer's protocol, twice as much chloroform (Thermo Fisher Scientific) as recommended (400 μL) was added to the tubes. The tubes were vortexed for another 10 min or until tissue was dissolved. The tubes were centrifuged at 10,000 × *g* for 10 min for phase separation. The top clear aqueous layer (350 μL) was harvested and combined with 350 μL of 70% ethanol (1:1) and applied to PureLink RNA columns (Thermo Fisher Scientific, Life Technologies; Cat #12183025). RNA was then purified following the manufacturer's protocol and incorporated the addition of DNase (Thermo Fisher Scientific, Life Technologies; Cat #12185010) to eliminate DNA contamination. RNA should be stored at −80°C. RNA concentrations were measured on a SpectraMax M2 spectrophotometer (Molecular Devices). Absorbance (A) values at 230, 260, and 280 nm were determined and are also an assessment of purity. The RNA samples should have an A260/A280 of ~2.0 and an A260/A230 of 2.0−2.2. Higher A260/A230 ratios may indicate organic compound (i.e., TRIzol)

contamination, and those samples should not be used. RNA samples should also be assessed by gel to visualize the 28-s rRNA and 18-s rRNA (Figure S4) or by microcapillary electrophoresis to determine the RNA integrity number (RIN) to confirm adequate quality and integrity.[33] After quantifying RNA, cDNA should be generated within 7 days to limit changes due to RNA degradation.

Human-specific GAPDH qRT-PCR

RNA was converted to cDNA using iScript (Bio-Rad; Cat #1708890) following the manufacturer's protocol. Briefly, 0.25 μg of RNA of each sample was added to 2 μL of 5× iScript reaction mix, and RNase-free water was added to 10 μL of total reaction volume and mixed with a pipette tip. The reactions (in 8-strip PCR tubes) were incubated in a thermal cycler following the manufacturer's protocol (5 min at 25°C, 20 min at 46°C, 1 min at 95°C, hold at 4°C optional). Notably, due to potential low levels of residual DNase contamination, it is important to store cDNA at −20°C, avoid long-term storage beyond 1 month, and keep cDNA samples on ice when being utilized in qRT-PCR reactions.

Before use in qRT-PCR reactions, the cDNA samples were diluted 1/10 in the 8-strip PCR tubes. With the use of a multichannel (helps reduce variability between samples due to pipetting error), 4 μL was dispensed into a 384-well qPCR plate (Bio-Rad). Subsequently, the SsoAdvanced Universal SYBR Green Supermix (Bio-Rad; Cat #172-5270) Master Mix containing 5 μL of the SsoAdvanced Universal SYBR Green Supermix and 1 μL of target primers (from 4 μM stock containing forward and reverse primers) was diluted to a final working concentration of 0.4 μM in the qPCR reaction, according to the manufacturer's protocol. 10 μL of total reaction was loaded per well in duplicate. The sequences for the human-specific and nonspecific mouse GAPDH primers were generated using the NCBI BLAST (Table S1). The qRT-PCR was performed on a CFX384 Touch Real-Time PCR Detection System (Bio-Rad; Cat #1855485). Table S4 outlines the reaction conditions. Other real-time PCR detection systems can be utilized.

To test the specificity of the primers and determine the efficiency of the primers (shown in Figure 3; Table S1), we generated cDNA as described above from 0.25 μg of RNA purified from MDA-MB-231 cells or from naive mouse lung (left lobe). The starting 1/10 diluted cDNA was then serially diluted 2-fold. Since we used total RNA/cDNA from MDA-MB-231 cells or mouse lungs as the template material with unknown copy numbers of human GAPDH or mouse GAPDH, we set the starting material as the arbitrary default value in Bio-Rad's CFX Manager software (1,000,000). The subsequent 2-fold dilutions were then set at 500,000; 250,000; 125,000; 62,500; 31,250; 15,625; and 7,812.5. The primer standard curves were then generated by plotting the Ct values versus the $\log_{10}$ of the arbitrary set starting material amounts of each sample.

Statistical analyses

All statistical analyses were performed using GraphPad Prism 8. In all cases where two experimental conditions are compared, an unpaired Student's t test was performed. The data were assessed for normality by performing the D'Agostino & Pearson test using GraphPad Prism. If the data were not normally distributed, then a Mann-Whitney test was performed.

SUPPLEMENTAL INFORMATION

Supplemental Information can be found online at https://doi.org/10.1016/j.omtm.2020.12.010.

ACKNOWLEDGMENTS

Support was provided by grant funding to P.M. from the Canadian Institutes of Health Research (CIHR; PJT 162313). M.L.D. was supported by a CGS-D award from the CIHR, a Nova Scotia Health Research Foundation studentship, a Nova Scotia graduate scholarship, and a Killam Laureate scholarship. K.M.C. was supported by a CGS-D award from the CIHR, the DeWolfe Graduate Award from the Dalhousie Medical Research Foundation, and by a studentship from the Beatrice Hunter Cancer Research Institute and the Canadian Imperial Bank of Commerce.

AUTHOR CONTRIBUTIONS

M.L.D., C.A.D., D.B.J., and K.M.C. generated and analyzed the data. M.L.D., C.A.D., and P.M. wrote the paper and made the figures. P.M. conceived of the study. All authors reviewed and approved the manuscript.

DECLARATION OF INTERESTS

The authors declare no competing interests.

REFERENCES

1. Dillekås, H., Rogers, M.S., and Straume, O. (2019). Are 90% of deaths from cancer caused by metastases? Cancer Med. *8*, 5574–5576.
2. Lambert, A.W., Pattabiraman, D.R., and Weinberg, R.A. (2017). Emerging Biological Principles of Metastasis. Cell *168*, 670–691.
3. Koga, Y., and Ochiai, A. (2019). Systematic Review of Patient-Derived Xenograft Models for Preclinical Studies of Anti-Cancer Drugs in Solid Tumors. Cells *8*, 418.
4. Jenkins, D.E., Oei, Y., Hornig, Y.S., Yu, S.F., Dusich, J., Purchio, T., and Contag, P.R. (2003). Bioluminescent imaging (BLI) to improve and refine traditional murine models of tumor growth and metastasis. Clin. Exp. Metastasis *20*, 733–744.
5. Deroose, C.M., De, A., Loening, A.M., Chow, P.L., Ray, P., Chatziioannou, A.F., and Gambhir, S.S. (2007). Multimodality imaging of tumor xenografts and metastases in mice with combined small-animal PET, small-animal CT, and bioluminescence imaging. J. Nucl. Med. *48*, 295–303.
6. Ni, Y., Wang, H., Chen, F., Li, J., DeKeyzer, F., Feng, Y., Yu, J., Bosmans, H., and Marchal, G. (2009). Tumor models and specific contrast agents for small animal imaging in oncology. Methods *48*, 125–138.
7. Stella, G.M., Kolling, S., Benvenuti, S., and Bortolotto, C. (2019). Lung-Seeking Metastases. Cancers (Basel) *11*, 1010.
8. Taus, L.J., Flores, R.E., and Seyfried, T.N. (2017). Quantification of metastatic load in a syngeneic murine model of metastasis. Cancer Lett. *405*, 56–62.
9. Fernando, W., Coyle, K., Marcato, P., Vasantha Rupasinghe, H.P., and Hoskin, D.W. (2019). Phloridzin docosahexaenoate, a novel fatty acid ester of a plant polyphenol, inhibits mammary carcinoma cell metastasis. Cancer Lett. *465*, 68–81.
10. Fiebig, H.H., Maier, A., and Burger, A.M. (2004). Clonogenic assay with established human tumour xenografts: correlation of in vitro to in vivo activity as a basis for anticancer drug discovery. Eur. J. Cancer *40*, 802–820.
11. Franken, N.A.P., Rodermond, H.M., Stap, J., Haveman, J., and van Bree, C. (2006). Clonogenic assay of cells in vitro. Nat. Protoc. *1*, 2315–2319.

12. Weng, D., Penzner, J.H., Song, B., Koido, S., Calderwood, S.K., and Gong, J. (2012). Metastasis is an early event in mouse mammary carcinomas and is associated with cells bearing stem cell markers. Breast Cancer Res. *14*, R18.

13. Yang, S., Zhang, J.J., and Huang, X.-Y. (2012). Mouse models for tumor metastasis. In Rational Drug Design, Y. Zheng, ed. (Humana Press), pp. 221–228.

14. DuPré, S.A., Redelman, D., and Hunter, K.W., Jr. (2007). The mouse mammary carcinoma 4T1: characterization of the cellular landscape of primary tumours and metastatic tumour foci. Int. J. Exp. Pathol. *88*, 351–360.

15. Nielsen, B.S., Lund, L.R., Christensen, I.J., Johnsen, M., Usher, P.A., Wulf-Andersen, L., Frandsen, T.L., Danø, K., and Gundersen, H.J.G. (2001). A precise and efficient stereological method for determining murine lung metastasis volumes. Am. J. Pathol. *158*, 1997–2003.

16. Mathenge, E.G., Dean, C.A., Clements, D., Vaghar-Kashani, A., Photopoulos, S., Coyle, K.M., Giacomantonio, M., Malueth, B., Nunokawa, A., Jordan, J., et al. (2014). Core needle biopsy of breast cancer tumors increases distant metastases in a mouse model. Neoplasia *16*, 950–960.

17. Mendoza, A., Gharpure, R., Dennis, J., Webster, J.D., Smedley, J., and Khanna, C. (2013). A novel noninvasive method for evaluating experimental lung metastasis in mice. J. Am. Assoc. Lab. Anim. Sci. *52*, 584–589.

18. Borin, T.F., Shankar, A., Angara, K., Rashid, M.H., Jain, M., Iskander, A., Ara, R., Lebedyeva, I., Korkaya, H., Achyut, B.R., and Arbab, A.S. (2017). HET0016 decreases lung metastasis from breast cancer in immune-competent mouse model. PLoS ONE *12*, e0178830.

19. Zhang, H., Yu, Y., Zhou, L., Ma, J., Tang, K., Xu, P., Ji, T., Liang, X., Lv, J., Dong, W., et al. (2018). Circulating tumor microparticles promote lung metastasis by reprogramming inflammatory and mechanical niches via a macrophage-dependent pathway. Cancer Immunol. Res. *6*, 1046–1056.

20. Liu, H., Patel, M.R., Prescher, J.A., Patsialou, A., Qian, D., Lin, J., Wen, S., Chang, Y.F., Bachmann, M.H., Shimono, Y., et al. (2010). Cancer stem cells from human breast tumors are involved in spontaneous metastases in orthotopic mouse models. Proc. Natl. Acad. Sci. USA *107*, 18115–18120.

21. Marcato, P., Dean, C.A., Liu, R.-Z., Coyle, K.M., Bydoun, M., Wallace, M., Clements, D., Turner, C., Mathenge, E.G., Gujar, S.A., et al. (2015). Aldehyde dehydrogenase 1A3 influences breast cancer progression via differential retinoic acid signaling. Mol. Oncol. *9*, 17–31.

22. Blaheta, H.J., Sotlar, K., Breuninger, H., Bueltmann, B., Rassner, G., Garbe, C., and Horny, H.P. (2001). Does intensive histopathological workup by serial sectioning increase the detection of lymph node micrometastasis in patients with primary cutaneous melanoma? Melanoma Res. *11*, 57–63.

23. Abt, M.A., Grek, C.L., Ghatnekar, G.S., and Yeh, E.S. (2016). Evaluation of lung metastasis in mouse mammary tumor models by quantitative real-time PCR. J. Vis. Exp. e53329.

24. Deng, W., McLaughlin, S.L., and Klinke, D.J. (2017). Quantifying spontaneous metastasis in a syngeneic mouse melanoma model using real time PCR. Analyst (Lond.) *142*, 2945–2953.

25. Jojovic, M., and Schumacher, U. (2000). Quantitative assessment of spontaneous lung metastases of human HT29 colon cancer cells transplanted into SCID mice. Cancer Lett. *152*, 151–156.

26. Ficorella, C., Martínez Vázquez, R., Heine, P., Lepera, E., Cao, J., Warmt, E., Osellame, R., and Käs, J.A. (2019). Normal epithelial and triple-negative breast cancer cells show the same invasion potential in rigid spatial confinement. New J. Phys. *21*, 1–32.

27. Tu, Y.F., Kaipparettu, B.A., Ma, Y., and Wong, L.J.C. (2011). Mitochondria of highly metastatic breast cancer cell line MDA-MB-231 exhibits increased autophagic properties. Biochim. Biophys. Acta *1807*, 1125–1132.

28. Bidola, P., Martins de Souza E Silva, J., Achterhold, K., Munkhbaatar, E., Jost, P.J., Meinhardt, A.L., Taphorn, K., Zdora, M.C., Pfeiffer, F., and Herzen, J. (2019). A step towards valid detection and quantification of lung cancer volume in experimental mice with contrast agent-based X-ray microtomography. Sci. Rep. *9*, 1325.

29. Haines, B.B., Bettano, K.A., Chenard, M., Sevilla, R.S., Ware, C., Angagaw, M.H., Winkelmann, C.T., Tong, C., Reilly, J.F., Sur, C., and Zhang, W. (2009). A quantitative volumetric micro-computed tomography method to analyze lung tumors in genetically engineered mouse models. Neoplasia *11*, 39–47.

30. Alcoser, S.Y., Kimmel, D.J., Borgel, S.D., Carter, J.P., Dougherty, K.M., and Hollingshead, M.G. (2011). Real-time PCR-based assay to quantify the relative amount of human and mouse tissue present in tumor xenografts. BMC Biotechnol. *11*, 124.

31. Campbell, J.P., Mulcrone, P., Masood, S.K., Karolak, M., Merkel, A., Hebron, K., Zijlstra, A., Sterling, J., and Elefteriou, F. (2015). TRIzol and Alu qPCR-based quantification of metastatic seeding within the skeleton. Sci. Rep. *5*, 12635.

32. Sleigh, J.N., Weir, G.A., and Schiavo, G. (2016). A simple, step-by-step dissection protocol for the rapid isolation of mouse dorsal root ganglia. BMC Res. Notes *9*, 82.

33. Fleige, S., and Pfaffl, M.W. (2006). RNA integrity and the effect on the real-time qRT-PCR performance. Mol. Aspects Med. *27*, 126–139.

OMTM, Volume *20*

Supplemental Information

Human-specific GAPDH qRT-PCR is an accurate and sensitive method of xenograft metastasis quantification

Margaret L. Dahn, Cheryl A. Dean, Diana B. Jo, Krysta M. Coyle, and Paola Marcato

Supplemental Figures:

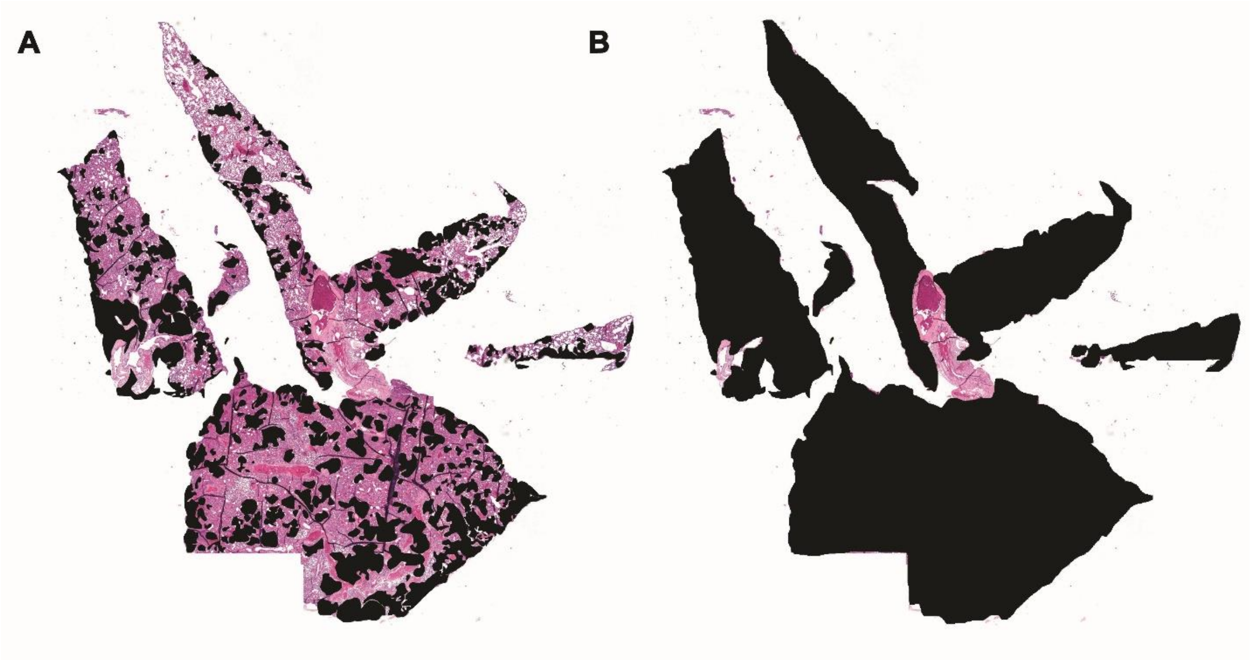

Supplemental Figure 1. Quantification of lung metastasis of H&E stained thin section using Image J. In the Image J software, the microscopically captured images were quantified of metastatic surface area by using the Freehand tool to circle and pixelate the metastatic foci **(A)**. The entire area of the lung was then selected and pixelated using the Freehand tool **(B)**, and % metastatic surface area for the thin section was calculated.

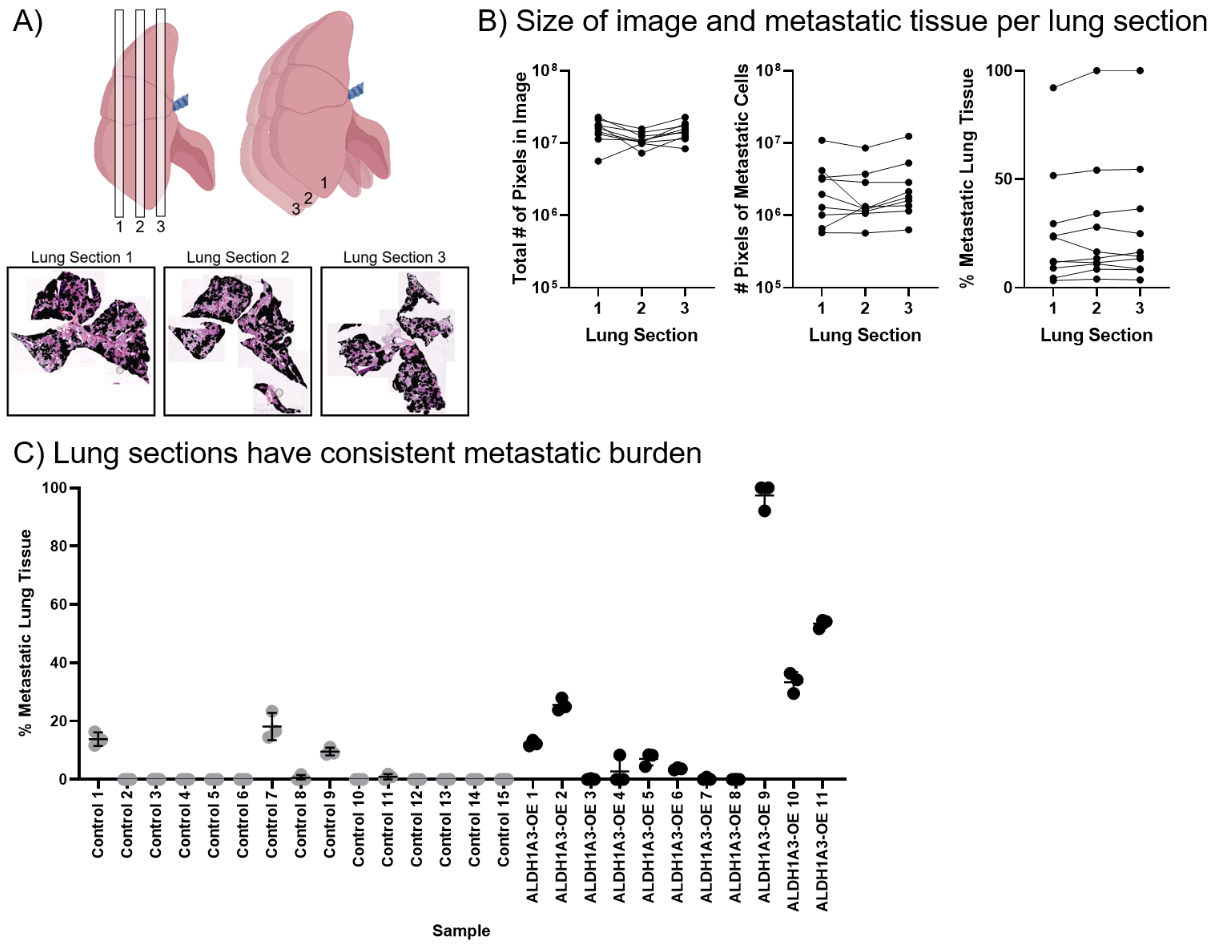

Supplemental Figure 2. Assessment of intrasample variability for metastasis quantification. **A)** Three longitudinal sections were taken of each lung along the sagittal (left) or coronal (right) plane from the first quarter, middle and last quarter of the right four-lobed lung. Representative images are shown for the three resulting sections. **B)** There is low intrasample variability when comparing metastatic burden between sections. The amount of tissue assessed in each section (as determined by number of pixels within the tissue) was similar between sections and between samples (left). The amount of metastatic tissue in each section (as determined by the number of pixels within metastatic regions) was consistent between sections taken from the same sample (middle). The portion of the lung which was composed of metastatic cells is consistent across

sections taken from the same sample (right). **C)** The mean % metastatic lung tissue across the three sections for each sample; standard deviation bars.

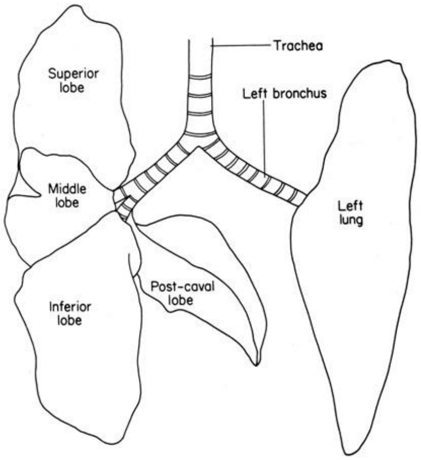

Supplemental Figure 3. Diagram of mouse lung. For consistency, the left lung lobe is always taken for the species-specific GAPDH RT-qPCR metastasis quantification method (the remainder is used for histological quantification).When mincing lung tissue in preparation for RNA extraction, utilizing two surgical blades simultaneously in a crisscross motion allows for easier/efficient mincing and dissociation of tissue.

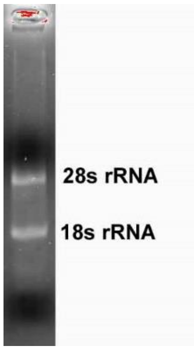

Supplemental Figure 4. RNA gel to confirm the integrity of purified RNA samples. The above image is a representative sample of purified mouse lung RNA utilizing the described method. The

visible crisp bands are 28s rRNA and 18s rRNA, which is indicative of intact RNA. Two ug of RNA (1 volume, 5µL) was added to three volumes (15µL) of RNA Sample Loading Buffer for NA electrophoresis, with ethidium bromide (50 µg/mL, Sigma-Aldrich) and heated at 65°C for 10 minutes and then kept on ice before being run on 1% agarose Tris Acetate-EDTA (TAE) gel. Molecular grade, RNase/DNase-free reagents were used and the gel apparatus was treated with RNaseZAP (Sigma-Aldrich) prior to casting.

Supplemental Tables:

Supplemental Table 1. Human and mouse GAPDH primer sequences and efficiencies.

Primer	*Forward*	*Reverse*	*Efficiency*
Mouse GAPDH (non-specific)	GCGAGACCCCACTAACATCA	GGCGGAGATGATGACCCTTT	107.4% with human cDNA, 109.8% with mouse cDNA
Human-specific GAPDH	TCAAGGCTGAGAACGGGAAG	CGCCCCACTTGATTTTGGAG	99.5%

Supplemental Table 2. ***In silico*** **analysis of human and non-specific mouse GAPDH primers.** Very wide parameters were set on NCBI Primer-BLAST for the theoretical RT-qPCR products that could be generated with a wide primer annealing temperature window (57 - 63°C) and large amplicon size range (70 - 1 000 bp) to capture any potential amplification, no matter how unlikely. Primers were run again both human and mouse reference transcriptome (NCBI RefSeq Database).

Primer	*Comparison Reference Transcriptome*	*Complementary Targets Identified*
Non-Specific mouse	Human	• No complementary sequences identified
	Mouse	• 100% fidelity for GAPDH TV1 and 2 and predicted TV1<br>• 4 mismatches on each primer to Kntc1 variants<br>• 1 mismatch on F primer to LOC115487111
Human-specific	Human	• 100% fidelity for GAPDH TV1-4<br>• 2 mismatches on F for GAPDH TV7
	Mouse	• 5 mismatches F, 4 mismatches R for MPRIP

Supplemental Table 3. Procedure for Infiltration (paraffin embedding of fixed lung tissue).

Note: All dehydration, hydration, and xylene steps are conducted in the hood for proper air ventilation. Once lung has been formalin fixed and washed in 70% ethanol, the tissue is ready to be processed for paraffin embedding. Tissues are first dehydrated, cleared with xylene and infiltrated (embedded) into paraffin wax, as shown in Supplemental Table 2. Tissues are embedding in paraffin wax using embedding rings, then placed at 4°C for 15 minutes to solidify.

DEHYDRATION	
70% ethanol	1.5 hr
95% ethanol	1.5 hr
95% ethanol	1.5 hr
100% ethanol	1.5 hr
100% ethanol	1.5 hr
100% ethanol	1.5 hr
CLEARING	
50:50 (100% ethanol:xylene)	1 hr
Xylene	1 hr
Xylene	1 hr
INFILTRATION (EMBEDDING)	
Paraffin wax (Tissue Prep, melting point 56-57C)	1 hr
Paraffin wax	1 hr

Supplemental Table 4. Procedure for H&E staining of fixed thin sections.

Note: Slides containing paraffin sections placed in a glass slide holder. Slides are first deparaffinized, then rehydrated in ethanol. Slides are then deionized in ddH_2O before H&E staining. Slides are then dehydrated in ethanol and xylene, before a cover slip is placed on top of the sections using Permount (xylene based).

DEPARAFFINIZE and REHYDRATE

Xylene	3 x 5 minutes
100% ethanol	3x 5 minutes
95% ethanol	2 x 5 minutes
70% ethanol	2 x 5 minutes

HEMATOXYLIN and EOSIN STAINING

Hematoxylin	1 x 60 dips
H2O (tap water)	1 x until excess stain is removed
Scott's H2O	1 x 30 dips
H2O (tap water)	1 x 10 dips
Eosin	1 x 20 dips
70% ethanol	1 x 30 dips
70% ethanol	1 x 30 dips

DEHYDRATION

70% ethanol	2 x 2 minutes
95% ethanol	2 x 2 minutes
100% ethanol	3 x 2 minutes
Xylene	3 x 2 minutes

Supplemental Table 5. qPCR reaction steps and temperatures.

qPCR CFX384 Steps		
1	95.0°C	0:30
2	95.0°C	0:05
3	60.0°C	0:05
	+ Plate Read	
4	Go To 2	40 more times
5	65.0 to 95.0°C	Increment 0.5°C for 0:05
	+ Plate Read	
End		
